# Measurement of Fracture Strength of Zirconia Dental Implant Abutments with Internal and External Connections Using Acoustic Emission

**DOI:** 10.3390/ma12122009

**Published:** 2019-06-23

**Authors:** Suet Yeo Soo, Nikolaos Silikas, Julian Satterthwaite

**Affiliations:** 1 Centre for Restorative Dentistry, Faculty of Dentistry, The National University of Malaysia, Selangor 43600, Malaysia; 2 Dentistry, School of Medical Sciences, University of Manchester, Manchester M13 9PL, UK; nikolaos.silikas@manchester.ac.uk (N.S.); julian.satterthwaite@manchester.ac.uk (J.S.)

**Keywords:** fracture strength, implant abutments, internal connection, external connection, acoustic emission

## Abstract

The aim of the study was to investigate the fracture behaviour of four different groups of zirconia abutments with internal and external connections: (A) Astra Tech ZirDesign™ abutment on Astra Tech Implants, (B) Procera^®^ Esthetic abutment on Nobel Biocare MK III Groovy Implants, (C) IPS e.max^®^ on Straumann Implants, and (D) ZiReal^®^ Posts on Biomet 3I implants. The load was applied on the assemblies using a Zwick universal testing machine: the initial and final failure loads and amplitude were recorded using acoustic emission technique. Mean initial and final failure force was found to be significantly different in each group (P < 0.001). IPS e.max^®^ Straumann abutments exhibited the highest resistance to final fracture force compared to other abutment types. Acoustic emission can be used as one of the methods to detect fracture behaviour of implant abutments. There were no significant differences in fracture loads between the internal and externally connected zirconia abutments studied. However, externally connected abutments demonstrated screw loosening and some deformations.

## 1. Introduction

Restoration of oral function and aesthetics has led to the increase in the use of dental implants over conventional restorative modalities [1]. Dental implants may provide multiple advantages including bone preservation, improved retention, better survival and optimisation of aesthetics and phonetics [2]. Different types of materials can be used to construct implant abutments. Metal abutments may have compromised aesthetics as greyish discolouration may be shined through on thin peri-implant mucosa [3]. Hence, more aesthetic zirconia ceramic abutments have gained popularity [4]. Zirconia implant-abutment connections can be classified into external and internal connections [5].

Although the success rates of dental implants have been increasing in recent years, failure of dental implants has also been reported [6]. They can largely be divided into biological, mechanical and aesthetic [7]. Mechanical failure could involve any part of the dental implant or abutment assembly. This is most common in their interface due to the concentration of torque and stress at this region [8]. Although ceramic abutments are preferred in some situations due to their aesthetic advantages, they are more brittle and less resistant to loads compared to traditional abutments, contributing to their failure due to crack formation and fracture [9]. At its initiation, microscopic cracks (initial fracture) form on the surface and these then propagate into a catastrophic fracture (final fracture) of the implant’s mechanism [8].

Multiple tests have been carried out to investigate the force required to induce a catastrophic fracture in an implant system [8,10]. Whilst universal loading machines generally provide the load, the detection of failure patterns can be performed via various modalities, for example scanning electron microscopy has been used to observe the fracture resistance on implant-supported metal ceramic crowns [10], and finite element analysis (FEA) has been used to mimic the behaviour of a material under loading stress [11]. The disadvantage of FEA is that it is based on mathematical models and the adoption of numerous assumptions. Materials used in the modelling are assumed to be homogenous, linearly elastic and isotropic and thus do not reflect their real-life behaviours [12].

Acoustic emission (AE) techniques have been employed to investigate the mechanical properties of biomaterials [13] by recording pressure waves generated during elastic deformation, micro-cracking, inter-crystal fracture and impurity fracture [14]. Although the final fracture forces of ceramic abutments have been well documented, little is known about the initiation of failure. Hence, the aim of this study was to investigate the initial and final fracture behaviour of different zirconia abutments with internal and external connections using acoustic emission (AE) technique.

## 2. Materials and Methods

### 2.1. Material Preparation

**Figure 1:**
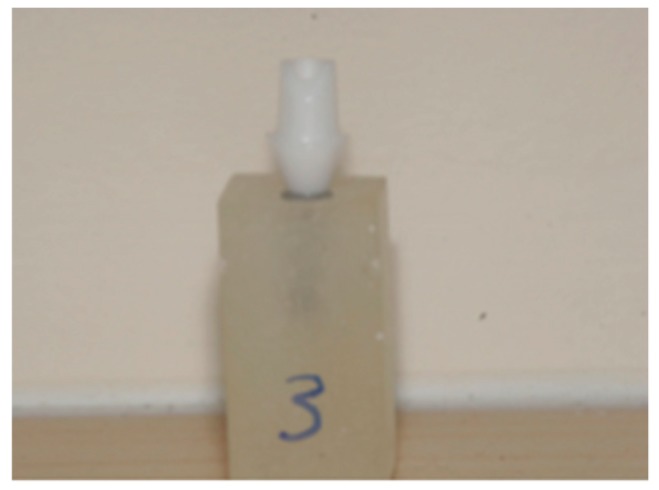
Implant-abutment assembly mounted on acrylic blocks.

Acrylic blocks were fabricated from preformed templates (Skill Bond Repair Acrylic). A master template was used to produce 60 acrylic blocks with dimensions of 20 mm × 10 mm × 10 mm. Four different types of the implant-abutment groups with external and internal connections (Table 1) were mounted in the acrylic blocks (Figure 1). The implant/abutment screw/abutment combination is hereafter referred to as the assembly.

Pilot testing for each implant was undertaken to ensure that appropriate size holes were drilled to produce an insertion torque of approximately 25 N cm (4.0 mm and 4.1 mm diameter implants were used with 3.7 mm and 3.8 mm diameter holes being most appropriate for the respective implant size). For each implant group, 14 blocks (comprising implant and abutment) were used for the investigations. A template was used to produce holes in the same location on each acrylic block. The depths were calibrated accordingly with a depth gauge, using a Vertical Drill Press (Kerry Drill Master, Kerrys (Engineering) LTD., Derbyshire, United Kingdom). The implants and abutments were torqued into the acrylic blocks as per manufacturer instructions. 

### 2.2. Universal Loading Machine

Load was applied on the assemblies using a Zwick Z020 universal testing machine (Zwick GmbH & Co. KG, Ulm, Germany) at 30 degrees off the vertical axis: The assemblies were mounted on a jig to ensure accurate positioning. A 4 mm stainless steel hemisphere was used to apply off axial load from the loading machine. A thin layer (0.1 mm) of Mylar film was inserted between the stylus and abutment. This was to prevent inadvertent surface damage by the loading stylus on the zirconia abutment and to further control loading. Loading was performed at a crosshead speed of 0.5 mm/min. Data was transmitted to an adjacent computer for analysis using Test Xpert (Zwick GmbH & Co. KG, Ulm, Germany), graphically presenting the data as Load (N) against time elapsed (s). 

### 2.3. Acoustic Emission

A 2-channel acoustic emission (AE) system (Physical Acoustics Corporation, Princeton Junction, NJ, USA) was used for the recording of acoustic emissions during loading. Although the apparatus contains two sensors, only one was utilised due to the size of the specimens. The sensor contained a transducer that converted acoustic waves into electrical signals. The AE sensor was approximated on the lateral surface of the zirconia abutment with a separating medium (Vaseline petroleum jelly). This was to allow optimal transmission of energy to the sensor. These were transmitted through two preamplifiers, of 40 dB gain and band pass of 100 kHz to 2 MHz. A specialised program, AE Win (Physical Acoustics Corporation, Princeton Junction, NJ, USA), allowed real time signal analysis. 

The data was graphically represented where the amplitude of acoustic signals (dB), counts (number of hits) and absolute energy (in AttoJoules) were plotted against test time(s). The collection of AE signals commenced once the loading hemisphere made contact with the abutment. As the loading progressed, acoustic signals were continuously collected and the data was collected. The experiments stopped when failure of the abutments or implants were observed.

In accordance with a previous study conducted in the same department and setting [14], 45 dB was selected as AE threshold amplitude (to avoid electrical and mechanical noises). Signals released above this level were deemed to indicate failure indication.

### 2.4. Statistical Analysis

Values of force and resultant amplitudes were extracted for all specimens. A statistical software package Graph Pad Prism (Version 5.03, Graph Pad Software Inc., San Diego, CA, USA) was used for analysis. Mean values were calculated.

Paired t-tests were conducted to detect significant differences between the initial and final fracture force and initial and final fracture amplitude in each group. One-way ANOVA and Bonferroni Post-Hoc tests were conducted to find statistically significant differences in the initial and final fracture forces between different groups. The level of statistical significance was set at p = 0.05.

## 3. Results

**Figure 2:**
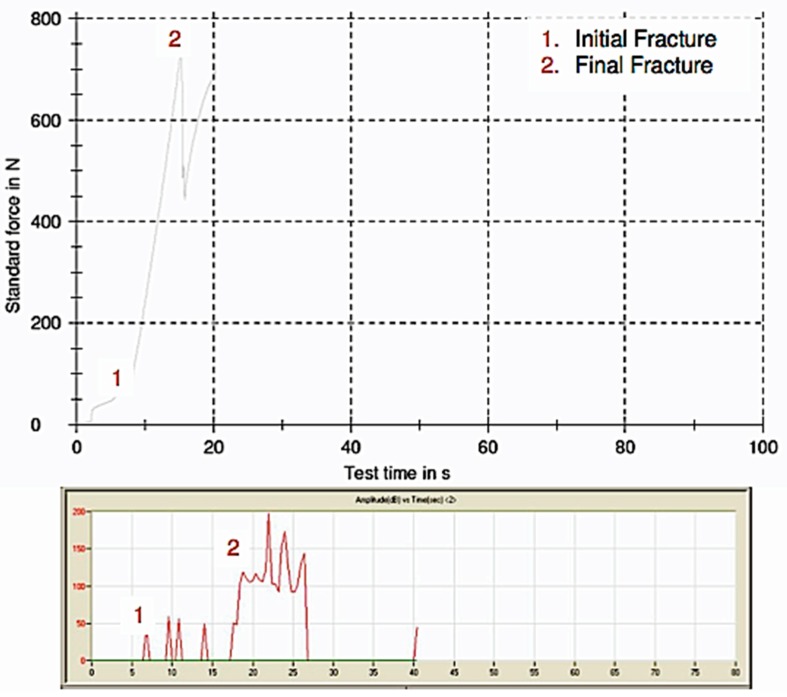
Graphs of the load-time relationship and amplitude-time relationship produced by the Zwick and AE sensor respectively.

Initial and final fracture points can be clearly identified from Figure 2. There was no observable fracture of the acrylic blocks. 

**Figure 3:**
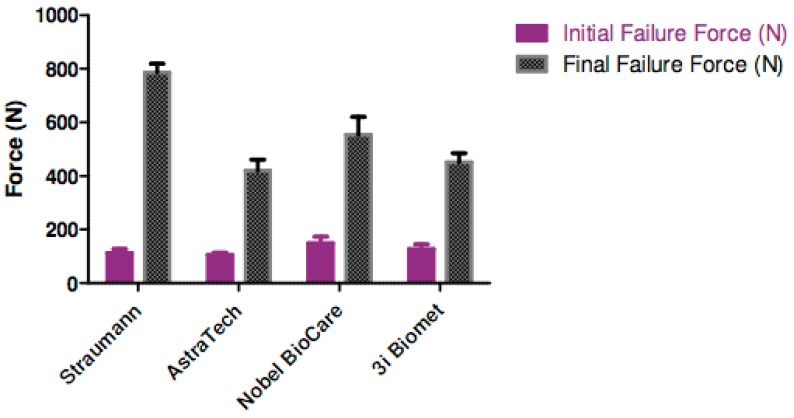
Comparison of initial and final failure force (N) of the assembly specimens.

Nobel Biocare/Procera^®^ aesthetic abutment had the highest mean resistance to initial fracture force, whilst Straumann/IPS e.max^®^ abutment had the highest mean resistance to catastrophic failure (Figure 3). From Table 2, comparing corresponding initial and final fracture force, there was a statistically significant difference in all the groups (p < 0.001). In terms of initial fracture force, there was no statistical difference between the groups (p = 0.25). However, Straumann IPS e.max^®^ abutment was shown to have a significantly higher final fracture force when compared to the rest (p < 0.001).

**Figure 4:**
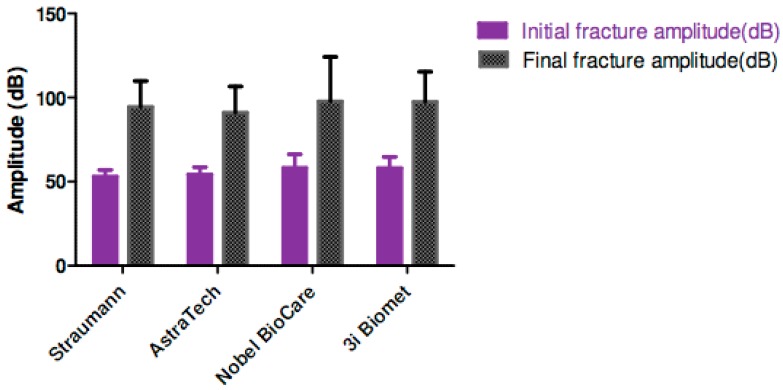
Initial and final fracture amplitude (dB) of the assembly specimens.

There was a significant difference between initial and final AE amplitudes in each group (p < 0.001) (Figure 4). 

**Figure 5:**
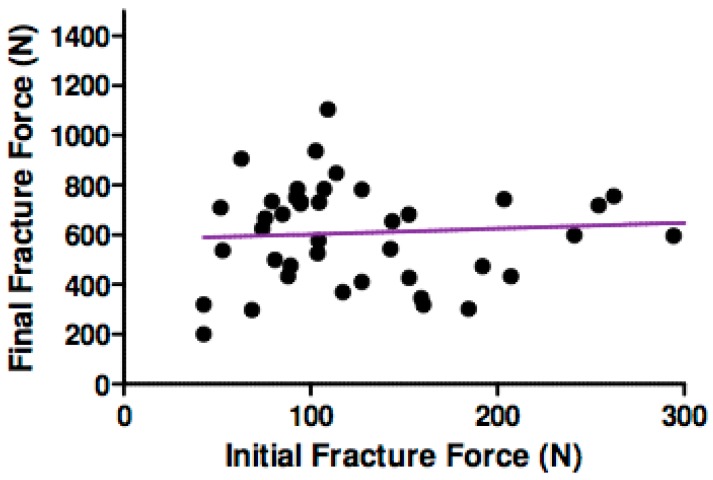
Graph correlation of initial and final fracture force (N).

Pearson correlation coefficient showed no significant relationship between initial and final force for all the assembly specimens in all groups, as illustrated in Figure 5 (p = 0.62, r = 0.006).

When the patterns of failure were explored, both internally- and externally-connected abutments fractured at the cervical aspect of the abutment adjacent to the abutment screw. In addition, abutment screws in the external connection groups experienced loosening, and in some samples, deformations were evident. There was no observable screw loosening in the internally connected assemblies.

## 4. Discussion

This study evaluated the efficacy of AE analysis in the assessment of initial and final fracture forces of zirconia abutments. Implant systems with differing connections were chosen to assess for any difference in behaviour (Table 1). The relative strength of internal and external connections to the underlying implants was also analysed (Table 2). The methodology was not adapted to the ISO 14801: 2007 norm of in vitro tests on implants. It was to investigate only the implant/abutment interface, and a full dynamic loading test was not applicable for the acoustic emission evaluation utilised in this study.

Acoustic emission is defined as “the phenomenon of transient elastic-wave generation due to a rapid release of strain energy caused by a structural alteration to a solid matter” [15]. In the event of structural changes, such as fracture, brittle and ductile materials result in high stress energy release in the form of pressure or sound waves. Therefore, AE techniques can be used to detect the initiation of failure and to determine the respective loading forces that form micro-cracks. As the loading force progressed, AE signal patterns revealed propagation of cracking and failure. This led to the determination of final failure forces of a specific abutment. 

Failure mechanism commences with the formation of microscopic cracks in ceramic dental materials. In a clinical setting, further cyclical loadings associated with mastication and parafunction propagate these cracks and lead to ultimate failure. Such microcracks could even be present during the manufacturing or pre-operative preparation phases [16]. As a result of this structural weakening, failure can be induced by forces lower than the experimental initial fracture forces. 

Previous studies have found the fatigue strength of zirconia abutments under static and dynamic loads, focussing on specific parameters such as height of abutments [8], preloading forces [17] and connection types [9]. In the present study, the final fracture loads (420–786 N) were comparable to those of above studies although the cited studies did not record initial fracture scores. It is worth noting that the initial fracture forces in this study were recorded to be 105–150 N and these readings were comparable to the maximal biting forces (144 N), higher than normal chewing force (50 N) [18].

The study of fracture resistance in ceramic abutments under load is of great significance [19]. During the implant dynamic loading process, highest torque and stress are found to be concentrated around the cervical region of implant abutments [8]. A fracture pattern mainly arising from the cervical part of the abutment in an area between the screw and the platform of the dental implant has been reported [20]. Our observations are consistent with the above studies; ceramic abutment fracture was the cause of failure in all our study specimens. Abutment screw fracture has been found to be a cause of failure [21]; however in that study, an abutment replica of stainless steel was used rather than the ceramic abutments in the present study.

It has been showed that an internal implant-abutment connection produces a more favourable load distribution [22] compared to external connections. Finite element analysis (FEA) has demonstrated that tensile forces induced by oblique loads are concentrated on the abutment screw threads [23], whereas internal connections with a tapered design share these stresses and protect the internal connected abutment screws from deformation. This finding has been further supported in a study that reported that internal connection via a secondary metallic component yielded the most superior strength [9]. Contradicting the above results, our study reported there was no significant difference in the overall performance of zirconia abutments with internal or external connections. The final fracture load of the Straumann RC Anatomical IPS e.max^®^ internally connected abutment was significantly higher (p < 0.001) compared to the other study specimens. Interestingly, the ZirDesign abutment had the lowest final fracture scores. Our results were supported by a study by Muhlemann at al. [24] that the type of connections (internal and external) only had a minor effect on the fracture loads in zirconia implants. It is worth noting that abutment screws in the external connection groups demonstrated screw loosening and some deformations.

In terms of assessment modality, AE was an effective method to detect initial and final fracture of ceramic abutments. For initial fracture detection, the mean AE amplitudes ranged from 53.0 to 58.1 dB and there was no statistically significant difference between those readings. For final fracture, the mean AE amplitudes ranged from 91.0 to 97.6 dB, and similarly, there was no statistically significant difference between the mean readings. In all the study groups, the final fracture mean amplitudes were significantly higher than the corresponding initial fracture amplitudes. Consequently, it can be concluded that the AE amplitude correlates well with the severity of the fracture from initial microcracks to catastrophic failure. Furthermore, there was no significant relationship between the AE amplitudes and the magnitude of the fracture loads, and this could further enhance the specificity of AE in fracture detection. Our results were supported by a previous study investigating the efficacy of AE in the detection of ceramic crown fractures [25].

AE as a technique to assess biomaterial failure is used increasingly; however, its use in dental biomaterials is rather limited. This study provides a unique insight on how to detect initial fracture in dental material and how to utilise AE technique in the detection of fractures in zirconia abutments. The ability to detect initial and final fracture values can be used to explain clinical failures. Clinically, these materials are more likely to fail due to application of continuous low loading values rather than due to a high value. 

## 5. Conclusions

AE has the ability to detect the initial and final fracture load of the implant system accurately. There was no significant difference in the fracture failure between internally and externally connected zirconia abutments. However, externally connected abutments demonstrated screw loosening and some deformations. 

## Figures and Tables

**Table 1:** Types of implant assembly used in this study.

Implant Manufacturer	Implant	Abutment	Connection Type
Astra Tech, Molndal, Sweden	4.0 mm (D) × 11 mm (L) Osseospeed	ZirDesign™ Abutment 3.5/4.0 4.5, 3 mm	Internal
Straumann, Basel, Switzerland	4.1 mm (D) RC × 10 mm (L) Bone Level SLA	Straumann RC Anatomical IPS e.max^®^ Abutment Straight, GH 3.5 mm, MO 0	Internal
Nobel Biocare, Goteberg, Sweden	4.0 mm (D) × 10 mm (L) RP MK III Groovy Implant	Procera^®^ Esthetic Abutment RP	External
Implant Innovations (Biomet 3i), Palm Beach, FL, USA	4.0 mm (H) × 10 mm (L) Certain	ZiReal^®^ Post 4.1 mm (D) × 4 mm (H)	External

**Table 2:** Means of the initial and final fracture force (N), percentage of the initial fracture force to its final fracture force and the respective acoustic amplitudes (dB) at the corresponding fractures.

Implant Assembly	Initial Fracture Force (N)	Final Fracture Force (N)	% of Initial to Final Force	Initial Fracture Amplitude (dB)	Final Fracture Amplitude (dB)
Astra Tech/ZirDesign™ Abutment (Internal connection)	105.3	420.1	25.1	54.3	91.0
Nobel Biocare/Procera^®^ Esthetic (External connection)	149.9	553.4	27.1	58.1	97.6
Straumann/Straumann RC Anatomical IPS e.max^®^ (Internal connection)	113.0	785.9	14.4	53.0	94.4
Implant Innovations (Biomet 3i)/ZiReal^®^ Post (External connection)	127.2	451.4	28.2	58.1	97.4
